# Phosphorus-solubilizing *Trichoderma* spp. from Amazon soils improve soybean plant growth

**DOI:** 10.1038/s41598-020-59793-8

**Published:** 2020-02-18

**Authors:** Laura Bononi, Josiane Barros Chiaramonte, Camila Cristiane Pansa, Marta Alves Moitinho, Itamar Soares Melo

**Affiliations:** 1 Laboratory of Environmental Microbiology, Brazilian Agricultural Research Corporation, EMBRAPA Environment, SP 340 Highway – Km 127.5, 13820-000 Jaguariúna, SP Brazil; 2 College of Agriculture “Luiz de Queiroz”, University of São Paulo, Pádua Dias Avenue, 11, 13418-900 Piracicaba, SP Brazil

**Keywords:** Applied microbiology, Soil microbiology

## Abstract

Acidic soils rapidly retain applied phosphorus fertilizers and consequently present low availability of this nutrient to plants. The use of phosphate-solubilizing microorganisms to help plant phosphorus (P) absorption is a promising sustainable strategy for managing P deficiencies in agricultural soils. *Trichoderma* strains have been one of the most studied filamentous fungi for improving the production and development of several crop species mainly due to their capability for symbiotic associations and their ability to control soil-borne plant diseases. Thus, this work sought to bioprospect *Trichoderma* strains from the Amazon rainforest capable of solubilizing/mineralizing soil phosphate and promoting soybean growth. Soybean plants inoculated with selected *Trichoderma* strains were cultivated in soil under greenhouse conditions and under a gradient of rock phosphate and triple superphosphate. As a result, 19.5% of the isolated *Trichoderma* strains were able to solubilize phosphate. In addition, those strains produced different organic acids during the solubilization process. *Trichoderma* spp. strains showed positive responses in the promotion of soybean growth—from 2.1% to 41.1%—as well as in the efficiency of P uptake-up to 141%. These results reveal the potential of *Trichoderma* spp. from the Amazon biome as promising biofertilizer agents.

## Introduction

The high demand for fertilizers used in Brazilian agriculture is a result of the growing population, which necessitates an increase in food production^1^. Brazil is the second-largest supplier of food and agricultural products and expected to be the leading producer of food to meet global demand in the near future^2^. Thus, applications of fertilizer are a routine activity in agricultural production as an attempt to promote crop growth to increase productivity. The requirement of fertilizers in the field results in the accumulation of those inputs in soils and water and, therefore, environmental pollution, causing problems to human and animal health^1,3^. In the future, Brazilian agriculture has to identify alternatives to reduce its dependence on chemical fertilizers while at the same time functioning in a lucrative and more sustainable way^4^.

A range of nutrients is important for plant growth^5^, but the ones that limit agricultural production the most are nitrogen and phosphorus, which are important in the initial development of the plant^6,7^. Nitrogen fertilization in Brazil has decreased significantly with use of symbiotic associations with nitrogen-fixing bacteria^8,9^. However, Brazilian agriculture continues to depend on chemical phosphate fertilization^4^. The role of P in the plant is associated with three essential biochemical processes: energy production, respiration, and photosynthesis. P is also involved in enzymatic processes and is a component of nucleic acids and cell membranes^10–13^. Phosphorus is generally found in the lowest concentration in the soil, 0.01%, compared to 0.14% of nitrogen, mainly in tropical and subtropical regions^14,15^. Although there is a high amount of total phosphorus (P) in the soil, its low availability to plants is one of the main obstacles to agricultural productivity^1^. The amount of P absorbed by the crops varies from 10 to 40% of the total phosphate fertilizer applied to the soil^16^. This phenomenon is due to a high degree of reactivity that occurs between phosphorus and soil constituents, causing the fixation of phosphorus or its precipitation with soil particles, making it unavailable for plant absorption^17^.

In general, Brazilian soils present low phosphorus contents (0.03 mg available P.kg^−1^ of soil), requiring high applications of phosphate fertilizers to meet cultural demands^18^. The efficiency of the application of phosphate fertilizers to the soil varies from 10–25%, and the phosphorus accessible to microorganisms and plants provided by these fertilizers is very low^19^. Brazil is the fourth largest country in terms of fertilizer consumption. According to the National Association for the Diffusion of Fertilizers, imports of phosphate fertilizers and domestic production of phosphate inputs in 2017 increased by 56.2% and 33.7%, respectively, the largest increases in relation to nitrogen and potash fertilizers. The high import rate of these fertilizers strongly contributes to a negative deficit in the Brazilian trade balance.^20^. In the last twenty years, the application of phosphate fertilizers has surpassed the expansion of arable land because of the rapid fixation of phosphorus in the soil. Maize, soybean, and sugarcane are the crops that receive most of these phosphate fertilizers^4^. The fertility of tropical soils is generally dependent on a thin layer of organic matter associated with litter. Thus, soils are highly weathered and typically acidic, and the availability of nutrients depends primarily on nutrient cycling^21,22^. The Amazon rainforest is among the forests with the largest reserves of biodiversity^23^. The biodiversity of the Amazon rainforest is attributed to the high variability of niches within it, from dense forest to savannah, making Brazil among those countries with a large macro- and microbiological biodiversity^24^. A large number of micro-ecosystems, soil types and climate conditions are favorable for fungal soil communities, providing a constant degradation of forest biomass^25,26^, which makes the Amazon rainforest a substantial source of bioprospection from fungal-originating products.

The application of microorganisms as biofertilizers is a promising approach to assist in agricultural production; these applications have contributed to the growth of several crop species^1,27,28^, increase plant biomass and total P contents^29^ and participate in the cycling of P without affecting the environment. Agricultural and pasture soils are composed of larger communities of these microorganisms involved in the availability of P^10,30^. They are key to providing phosphorus that is retained in the soil to plants^1^ via the processes of mineralization and solubilization^31^. Sharma *et al*. (2013) reported the ability of fungi to occupy larger spaces and ranges within the soil than bacteria and to produce a range of organic acids that play a trivial role in the solubilization of inorganic phosphate. Within this context, we believe that the Amazon rainforest may be an excellent biome for the bioprospection of fungal strains capable of solubilizing/mineralizing insoluble phosphorus and making a portion of this nutrient pool available to plants. In this way, the Amazon rainforest can improve the growth and productivity of a wide variety of crop species.

Fungi of the genus *Trichoderma* pertain to the phylum Ascomycota, subdivision Pezizomycotina, class Sordariomycetes, order Hypocreales and family Hipocreaceae^32,33^. They are rapidly growing fungi in the culture medium, initially presenting colonies with the presence of white mycelium, which with development becomes cottony and compact with green tufts. They are cosmopolitan, found most in soils and have an important ecological function, because they participate in the decomposition and mineralization of plant waste, contributing to the availability of nutrients for the plants, interfering directly and indirectly in their growth^34,35^. *Trichoderma* is commonly studied for the control of soil phytopathogens and as a biofertilizer. *Trichoderma* have great prominence in studies^28,36^, due to their predominance in the rhizosphere of different plant species, role in the control of different phytopathogens, assistance in the promotion of plant growth via different mechanisms. Furthermore, easy isolation, rapid growth and ability to grow in different substrates, as well as their ability to produce an infinite amount of metabolites such as antibiotics and auxins^37^. Recent studies have reported the occurrence of *Trichoderma* in soils of the Amazon forest that have cellulolytic capabilities^38,39^, are endophytes of native tree species^40^ are producers of biosurfactants^41^ and are biological control agents^42,43^. Thus, our work sought to bioprospect *Trichoderma* strains from the Amazon rainforest with the ability to solubilize/mineralize phosphate and the potential to promote the growth of soybean plants, as the environment of the Amazon rainforest is highly dependent on microorganisms for fast nutrient cycling. With the application of *Trichoderma* strains in the soil, we sought to optimize the use of different sources of phosphates, as well as the quantity of these sources applied, to contribute to more sustainable agriculture and greater efficiency in the use of phosphorus sources.

## Results

### Selective isolation and selection of *Trichoderma* spp. capable of solubilizing phosphate *in vitro*

Phosphorus is the most important macronutrients in crop development and growth, and phosphate-solubilizing fungi play an important role in enhancing phosphorus availability for plants. A total of 251 isolates were obtained from Amazonian soils using the selective medium TSM for *Trichoderma*. The numbers of isolates per collection point are shown in Table 1. The isolates were preserved at the Collection of Microorganisms of Environmental and Agricultural Importance (CMAA) of EMBRAPA Environment, Jaguariúna, São Paulo, Brazil.

**Table 1:** Collection data.

Collection Point	Collection Time	Coordinates		Soil Temperature (°C)	Type of Soil	Number of *Trichoderma* isolates
		S	W			
1	April/2015	02°50′58,8″	59°24′52,2″	24,7	clay	39
2	April/2015	02°54′41,7″	59°02′26,6″	28	sandy	7
3	April/2015	03°00′45,4″	58°51′12,6″	25,3	sandy	10
4	April/2015	03°07′33,2″	60°00′22,9″	24,1	sandy	18
5	April/2015	03°12′35,8″	60°40′43,3″	25,9	sandy	17
6	April/2015	02°59′21,6″	60°53′36,0″	24	sandy	29
7	April/2015	02°51′36,5″	60°58′10,6″	26,2	sandy	18
8	April/2015	02°17′49,9″	60°02′37,7″	24,3	clay	20
9	April/2015	01°49′46,1″	60°07′55,0″	25,2	clay	24
10	April/2015	01°28′39,2″	60°15′10,0″	27,5	sandy	20
11	April/2015	01°28′52,4″	60°15′18,9″	25,1	clay	26
12	April/2015	01°56′52,5″	60°02′31,8″	26	clay	23
Total number of isolates						251

From: Phosphorus-solubilizing *Trichoderma* spp. from Amazon soils improve soybean plant growth.

To select the phosphate-solubilizing fungi, the clear zone was observed around the colonies of *Trichoderma* spp. isolates on solid NBRIP media. This effect occurs because, during their growth, the microorganisms use the phosphate present in the culture media. Of all *Trichoderma* spp. isolates screened, 49 showed potential for solubilizing phosphorus, with halos greater than 10 millimeters (Table 2). Of these isolates, eight with halos greater than 50 mm were selected for testing in NBRIP liquid media. The eight isolates presented a halo around the colony on PSM media (halos ranged from 5.3 to 10.7 millimeters in diameter), indicating the ability to mineralize organic phosphorus in the form of phytate.

**Table 2:** Phosphate solubilization and mineralization in solid NBRIP and PSM culture medium and organic acid production by *Trichoderma* spp.

Treatments	Solubilization/Mineralization			Average	Standard deviation	Organic Acid
	Halo size (mm)					
	1	2	3			
AMS 34.39	53	53	54	53.3	0.58	Latic Acid, Fumaric Acid
	10	10	12	10.7	1.15	
AMS 29.10	57	54	50	53.7	3.51	Ascorbic Acid, Gluconic Acid
	11	9	10	10	1.0	
AMS 1.43	56	50	58	54.7	4.16	Latic Acid
	6	5	5	5.3	0.58	
AMS 2.18b	65	57	49	57	8.0	D-Malic Acid
	7	15	9	10.3	4.2	
AMS 31.15	53	60	60	57.7	4.0	D-Isocitric Acid, Phytic Acid, Citric Acid
	12	9	10	10.3	1.5	
AMS 26.10	53	60	60	57.7	4.0	D-Malic Acid
	11	10	9	10	1	
AMS 2.18a	55	62	64	60.3	4.7	Ascorbic Acid, Gluconic Acid
	8	6	6	6.7	1.1	

From: Phosphorus-solubilizing *Trichoderma* spp. from Amazon soils improve soybean plant growth.

**Figure 1:**
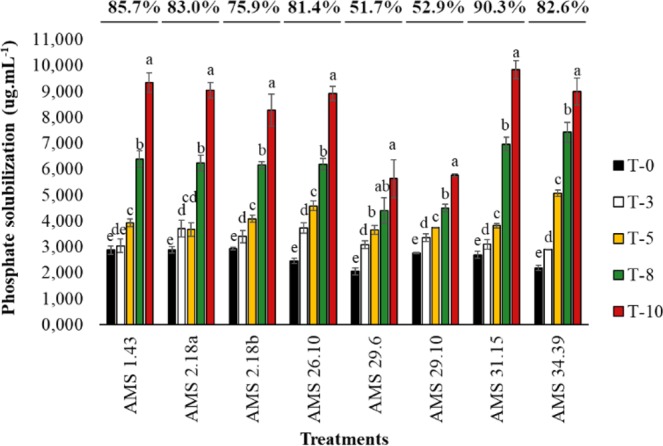
Values in percentage after the ten-day incubation period and µg.mL^−1^ of calcium phosphate solubilization (10 g.L^−1^) in liquid NBRIP medium by *Trichoderma* spp. during the ten-day incubation period. T-0 - first day of incubation, T-3 - third day of incubation, T-5 - fifth day of incubation, T-8 - eighth day of incubation, T-10 - tenth day of incubation. Different letters are significantly different according to Tukey test (P < 0.05). From: Phosphorus-solubilizing *Trichoderma* spp. from Amazon soils improve soybean plant growth.

The isolates AMS 31,15 (90,3%), AMS 1.43 (85.7%), AMS 2.18a (83.0%) and AMS 34.39 (82.6%) were also selected as fungi with potential for solubilization (Fig. 1). Of the four isolates of *Trichoderma* spp. with the best results, two (AMS 34.39 and AMS 31.15) did not inhibit the germination of soybeans, as determined via a culture used in an experiment in the greenhouse. For this reason, these two isolates were selected for bioassays in the greenhouse with soybean plants.

The eight selected isolates produced organic acids during the solubilization process. They produced lactic acid, fumaric acid, ascorbic acid, gluconic acid, d-malic acid, d-isocitric acid, citric acid, and phytic acid, as shown in Table 2.

### Impact of *Trichoderma* spp. and phosphate fertilizers on the development of soybean plant

**Figure 2:**
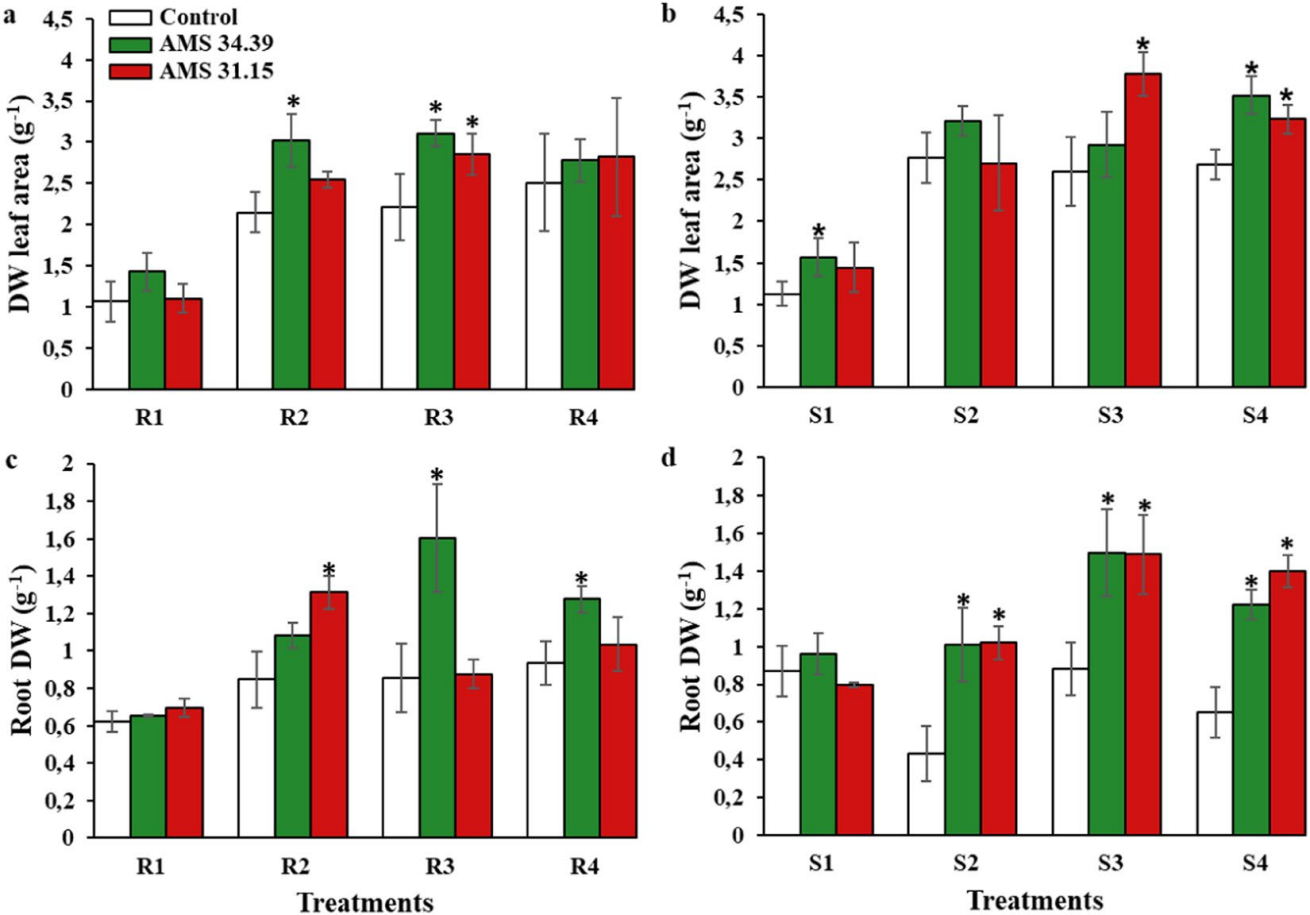
Average dry matter mass of the leaf area and root of soybeans grown in soils with different levels of P (1, 2, 3 and 4) and sources of phosphorus (R- Bayóvar Rock Phosphate and S- Triple Super Phosphate) in the presence of *Trichoderma* (green and red) and Control (white). (**a**) DW leaf area - Bayóvar Rock Phosphate, (**b**) DW leaf area - Triple Super Phosphate, (**c**) Root DW - Bayóvar Rock Phosphate and (**d**) Root DW – Triple Super Phosphate. Different letters are significantly different according to Scott Knott test (P < 0.05). From: Phosphorus-solubilizing *Trichoderma* spp. from Amazon soils improve soybean plant growth.

In the absence of a source of phosphorus applied to the soil (Level 1), that is, without the application of the sources of rock phosphate and super triple phosphate, the plants responded positively to the increase in aerial biomass in the three treatments applied (control, AMS 34.39 and AMS 31.15) (Fig. 2a,b). However, the combination of *Trichoderma* and phosphorus sources increased significantly at level 3 (P < 0.05) the biomass of soybean plants in rock phosphate (Fig. 2a). This difference was also observed in the super triple phosphate source at levels 3 and 4 about the control (Fig. 2b). In both sources of phosphate, the AMS 34.39 isolate showed a significant difference when compared to the control at the same level. The DW of the leaf area between the phosphorus application levels varied from 10.5–40.7% (AMS 34.39) and 2.1–41.1% (AMS 31.15) compared that of to the control treatment (without *Trichoderma*) for the Bayóvar rock phosphate and super triple phosphate sources. For roots, the DW varied 4.9–134.9% for AMS 34.39 isolate and from 0.9–137.2% for AMS 31.15. The efficiency in the absorption of phosphorus by the plants inoculated with the *Trichoderma* strains was also evaluated. Compared to the control (control, level 1) values, the treatment values ranged from 111.2–156.1% (AMS 34.39) and from 81.7–140.6% (AMS 31.15) (Table 3).

**Table 3:** Overview of the experimental design and percentage of P absorption efficiency and biomass improvement.

Treatments	P source	Level P	Efficiency P (%)	Biomass improvement (%)	
				Leaf	Root
Control	Rock phosphate	R1	**___**	**___**	**___**
AMS 34.39	Rock phosphate	R1	120.5	33.8	4.9
AMS 31.15	Rock phosphate	R1	81.7	3.6	11.8
Control	Rock phosphate	R2	**___**	**___**	**___**
AMS 34.39	Rock phosphate	R2	132.8	30.8	27.5
AMS 31.15	Rock phosphate	R2	111.5	10.5	3.5
Control	Rock phosphate	R3	**___**	**___**	**___**
AMS 34.39	Rock phosphate	R3	156.1	40.7	87.5
AMS 31.15	Rock phosphate	R3	140.6	34.8	53.3
Control	Rock phosphate	R4	**___**	**___**	**___**
AMS 34.39	Rock phosphate	R4	111.2	10.7	36.8
AMS 31.15	Rock phosphate	R4	90.7	12.5	11.1
Control	Triple Super Phosphate	S1	**___**	**___**	**___**
AMS 34.39	Triple Super Phosphate	S1	140.9	39.6	10.7
AMS 31.15	Triple Super Phosphate	S1	121.8	28.7	0.9
Control	Triple Super Phosphate	S2	**___**	**___**	**___**
AMS 34.39	Triple Super Phosphate	S2	121.5	10.5	134.9
AMS 31.15	Triple Super Phosphate	S2	87.5	2.1	137.2
Control	Triple Super Phosphate	S3	**___**	**___**	**___**
AMS 34.39	Triple Super Phosphate	S3	135.1	23.1	130.2
AMS 31.15	Triple Super Phosphate	S3	127.0	41.1	129.2
Control	Triple Super Phosphate	S4	**___**	**___**	**___**
AMS 34.39	Triple Super Phosphate	S4	127.8	22.9	39.0
AMS 31.15	Triple Super Phosphate	S4	119.4	18.9	59.1

From: Phosphorus-solubilizing *Trichoderma* spp. from Amazon soils improve soybean plant growth.

**Figure 3:**
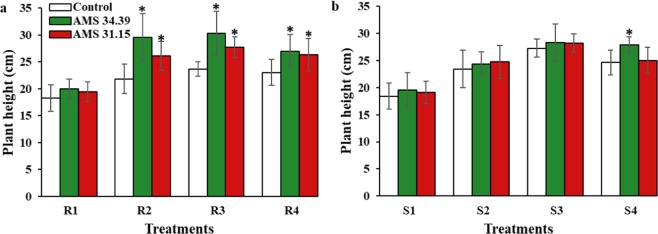
Average height of soybeans grown in soils with different levels of P (1, 2, 3 and 4) and sources of phosphorus (R- Phosphate of Bayóvar Rock and S- Triple Super Phosphate) in the presence of *Trichoderma* (green and red) and Control (white). (**a**) Bayóvar Rock Phosphate and (**b**) Triple Super Phosphate. Different letters are significantly different according to Scott Knott test (P < 0.05). From: Phosphorus-solubilizing *Trichoderma* spp. from Amazon soils improve soybean plant growth.

Significant differences (P < 0.05) were observed in the height of soybean plants when *Trichoderma* isolates were inoculated (Fig. 3a,b). This difference was most evident for the two isolates when P was applied (at levels 2, 3 and 4). It was also observed that the increase in plant height when inoculated with *Trichoderma* spp. compared with that of the controls was better with the source of Bayóvar rock phosphate.

**Figure 4:**
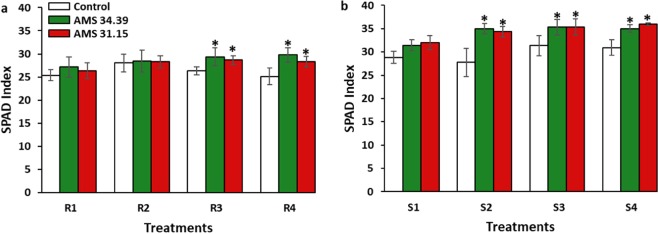
Average chlorophyll index of soybeans grown in soils with different levels of P (1, 2, 3 and 4) and sources of phosphorus (R- Bayóvar Rock Phosphate and S- Triple Super Phosphate) in the presence of *Trichoderma* (green and red) and Control (white). (**a**) Bayóvar Rock Phosphate and (**b**) Triple Super Phosphate. Different letters are significantly different according to Scott Knott test (P < 0.05). From: Phosphorus-solubilizing *Trichoderma* spp. from Amazon soils improve soybean plant growth.

The chlorophyll indices presented significant differences (P < 0.05) in response to the level-3 and level-4 P for the two phosphate sources and the two *Trichoderma* isolates. For the super triple phosphate source, the effect of the application of the *Trichoderma* strains and the increase in the phosphorus level was more promising (Fig. 4a,b).

### Enzymatic activities in the soil

**Figure 5:**
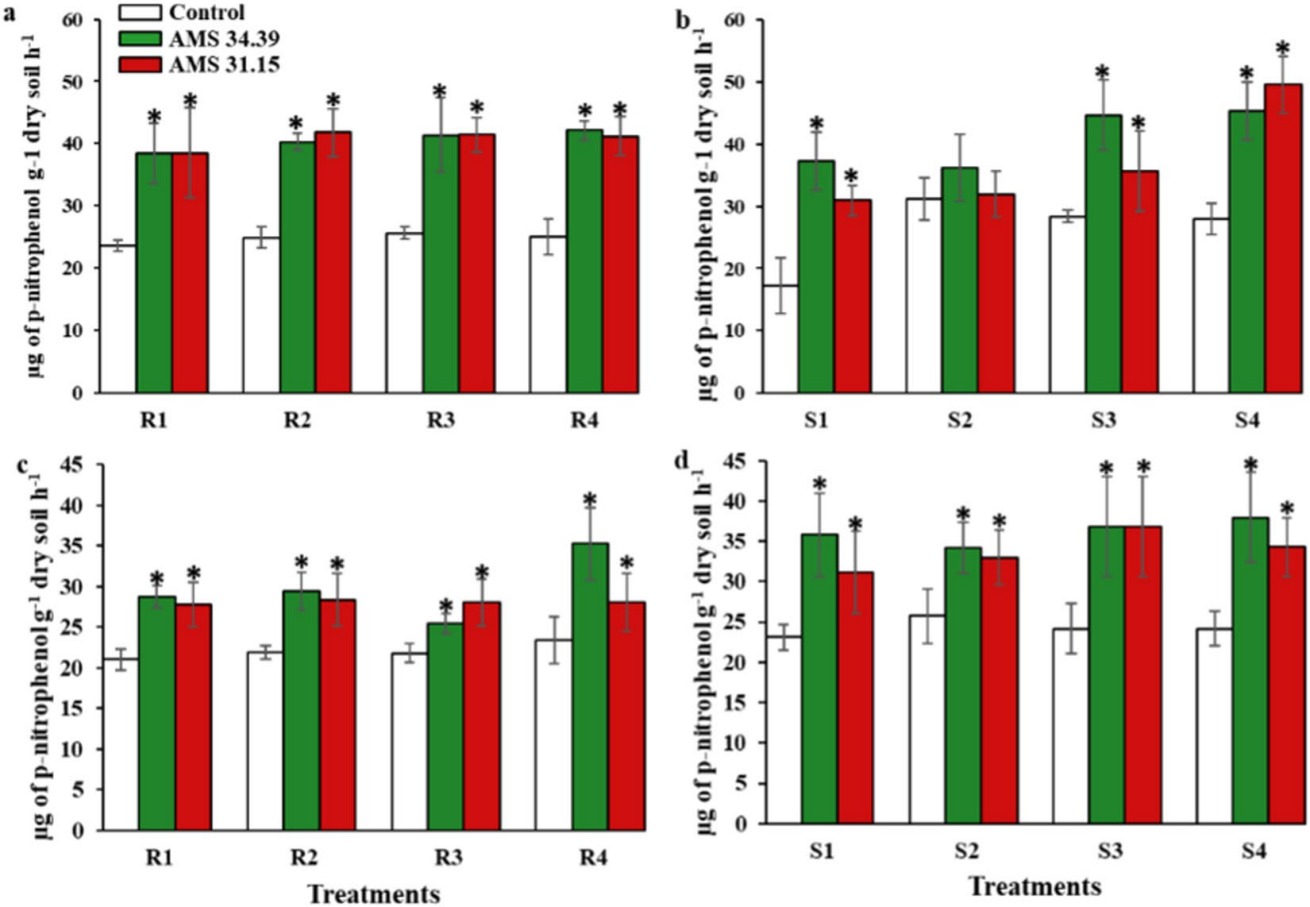
Average activity of acid and alkaline phosphatase in soils with different levels of P (1, 2, 3 and 4) and sources of phosphorus (R- Bayóvar Rock Phosphate and S- Triple Super Phosphate) in the presence of *Trichoderma* (green and red) and Control (white). (**a**) Acid Phosphatase - Bayóvar Rock Phosphate, (**b**) Acid Phosphatase – Triple Super Phosphate, (**c**) Alkaline Phosphatase - Bayóvar Rock Phosphate and (**d**) Alkaline Phosphatase – Triple Super Phosphate. Different letters are significantly different according to Scott Knott test (P < 0.05). From: Phosphorus-solubilizing *Trichoderma* spp. from Amazon soils improve soybean plant growth.

The activity of acid and alkaline phosphatases was similar between the isolates when the two sources of phosphorus were applied (Fig. 5a–d). In general, for the two phosphate sources studied, the AMS 34.39 and AMS 31.15 isolates showed significantly higher enzymatic activity (P < 0.05) than the controls at all levels of P applied to the soil.

**Figure 6:**
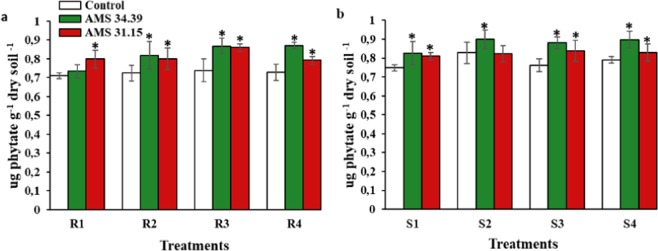
Average enzyme activity of phytase in soils with different levels of P (1, 2, 3 and 4) and sources of phosphorus (R- Bayóvar Rock Phosphate and S- Triple Super Phosphate) in the presence of *Trichoderma* (green and red) and Control (white). (**a)** Bayóvar Rock Phosphate and (**b**) Triple Super Phosphate. Different letters are significantly different according to Scott Knott test (P < 0.05). From: Phosphorus-solubilizing *Trichoderma* spp. from Amazon soils improve soybean plant growth.

There was a significant difference between the treatments with the presence of *Trichoderma* and those without inoculation with the fungus for activity phytase. The AMS 34.39 and AMS 31.15 isolates showed increases of up to 17% and 16%, respectively, with the source of Bayóvar rock phosphate. On the other hand, the use of triple superphosphate showed increases of up to 15% and 10% for the isolates AMS 34.39 and AMS 31.15, respectively (Fig. 6a,b).

The combination of *Trichoderma* with a phosphate fertilizer can be much more advantageous for the plant compared with their separate use in the field, that is, only *Trichoderma* applied without a source of phosphorus or a source of phosphorus without applications of *Trichoderma*.

## Discussion

In this study, *Trichoderma* isolated from soils of the Amazon rainforest demonstrated the potential for phosphate solubilization and increased soybean plant growth, highlighting the importance of the Amazon biome as a source of novel microbial stains with biotechnological importance. The solubilization process is caused by the release of organic acids and various enzymes, phosphatases, and compounds produced by microorganisms^44^. All this organic acid was already described as produced by *Trichoderma* strains^45^ (Table 2). Organic acids have great importance in the availability of phosphorus for the plant because they are capable of converting the phosphate present in the soil into di- or monobasic phosphates, which are readily available for absorption.

The fungus, applied in conjunction with a phosphorus source, promoted soybean plant growth (Fig. 3). The two phosphorus sources evaluated in this study showed higher positive effects when combined with the *Trichoderma* isolates than when applied alone. These effects were also P level dependent. Treatments involving different *Trichoderma* strains with beneficial attributes, including the promotion of plant growth and the biocontrol of phytopathogens, should be considered in the development of formulations. The positive effect of *Trichoderma* in the presence of a phosphorus source has also been reported by other authors^45–47^. Phosphorus that is readily available to the plant is a phosphate anion, a poorly mobile element in the soil and plants compared to other macronutrients. Thus, in addition to transforming organic phosphate into inorganic phosphate, *Trichoderma* helps to increase the root system, contributing to a greater region of nutrient absorption by the plant^48^. Few reported on the mechanisms of plant*-Trichoderma* interaction in promoting growth. Possibly one of the mechanisms involved in root development is due to acidification of the site with the presence of *Trichoderma*. This process results in the early development of the roots, which after the first days of development occurs an inhibition of the primary root and consequently the development of secondary roots^37,49^, as a mechanism to escape the acidification of the medium. According to Cornejo, *Trichoderma* enhances the lateral roots instead of the formation of new roots. Many authors have reported that during the process of P solubilization, the pH of the medium becomes acidified, probably due to the production of organic acids^30,50–52^. Thus, a correlation between the decrease in pH and the increase in P solubilization influences the biomass increase of the lateral roots^12^ and consequently increases the surface of P absorption by plants. Tandon (2019) demonstrated that by alkalinizing the medium in the phosphorus solubilization process, mycelial production and phosphatase activity by *Trichoderma* decreased significantly, which contributes to the importance of pH in the phosphorus solubilization process^53^. Combined with another mechanism that can be important in the formation of the root system is the production of metabolites, such as auxins and ethylene, produced by a range of *Trichoderma* species^49,54^.

The rock phosphate, being an insoluble phosphate, induces a higher secretion of phosphatases, for example, which facilitates the release of phosphorus to the plant promoting growth^55^. The mechanisms of P solubilization differ not only between fungal isolates but also between the phosphorus sources applied. Triple superphosphate has a higher content of soluble P available to the plant than does rock phosphate, considering that much of it is adsorbed to soil colloids. The microbial activity when rock phosphate is applied is higher because a greater amount of phosphate needs to be mineralized. The results obtained in this study showed the phosphate solubilization potential of two strains of *Trichoderma* spp. It is important to emphasize the use of rock phosphate, which has a relatively slow release of phosphorus in the soil, in addition to being a cheaper source because it requires a relatively low amount of manufacturing^56^. One of the major problems with the application of rock phosphate is that because it is slowly released, crops tend to have low yields in the initial few years. With the combined application of *Trichoderma* as presented in this paper, the response of the plants was positive (Fig. 2, Table 3). This joint application presents great importance for agriculture because there is relatively little expenditure with the use of rock phosphate and because the permanence of rock phosphate in the soil is greater than that of triple superphosphate, which is readily used; finally, with the *Trichoderma*, production can be relatively high.

In this work, The performance of *Trichoderma* spp. isolates were better presented in the application of phosphorus at level 3, especially with the AMS strain 34.39. Thus, the application of phosphorus could be in a smaller amount and with better efficiency when using together a *Trichoderma* strain (Fig. 2). For example, the phosphate level 3 applied represents the average productivity of the soybean crop. When applying AMS 34.39 isolate at level 3, we observed increases of 40.7% and 23.1% in response to the sources of Bayóvar and super triple rock phosphate, respectively (Table 3). When comparing the biomass values for the same strain of *Trichoderma* (AMS 34.39) combined with phosphorus at level 4, which is equivalent to the high productivity of the crop, it showed increases of 10.7% and 22.9% for the same sources of phosphorus applied. Thus, when applying 70 kg ha^−1^ of phosphate (level 3) the result was better for the biomass of soybean plants than the application equivalent to 90 kg ha-1 (level 4).

The low concentration of phosphorus in the soil reflects a decrease in ATP and NADPH production and the expression of genes related to photosynthesis^57^. Thus, these decreases are reflected in the chlorophyll index because it is an indication of photosynthetic pigments^58^. Therefore, the application of a phosphate near the *Trichoderma* may have reflected in the production of ATP in the plant, as well as in the expression of genes associated with photosynthesis, responding to the increase in chlorophyll in the results obtained (Fig. 4). Triple superphosphate, a readily available source in the soil, presented the most promising result because the analysis was performed twenty days after the planting of the crop, a result that was already expected. Some authors have demonstrated the increase on chlorophyll level due the presence of *Trichoderma* on different cultures as cucumber, wheat, soybean and lettuce plants^59–62^.

Some factors are involved in the process of phosphatase production by *Trichoderma*, such as the presence of an inorganic phosphate is essential for a better secretion of phosphatases, and it has been reported that the nature of the phosphate source linked to the solubilization process also interferes in the activity^52,55,63,64^. One of the mechanisms of action of *Trichoderma* for nutrient supplementation of plants is via the production of phosphatase enzymes. Some authors have already described the activity of this fungus in terms of its production of these enzymes^47,48,52,65^. The activity of phosphatases is reported mainly at sites where there is an absence of inorganic phosphorus^52^. In a study by Naik *et al*. (2013), acid phosphatase activity was higher for *Trichoderma* than for the other two fungi studied: *Aspergillus* and *Penicillium*^12^. The genus *Trichoderma* has been reported for its high phytase activity, which releases available phosphorus in the soil^30,46,66^. The results obtained in this study corroborate those found by those authors (Fig. 6a,b). The high association with the solid phase of the soil makes the phosphorus bound to phytate available in low quantities, limiting its absorption by plants^67^. Thus, phosphate fertilizers constitute the most soil-applied fertilizers to achieve good productivity. Many factors can interfere with the efficiency of phosphate-solubilizing microorganisms, such as the preparation of the inoculant, the form of application to the soil and the place where it is applied^51^. In addition, Garcia Lopes (2017) demonstrated that the type of soil may be related to the activity of microorganisms^68^. The concentration of P applied to the different soil was not affected, but its efficiency was affected by the physical and chemical properties of the soil^69,70^.

The efficiency of microorganisms that assist in the availability of P in the soil is correlated with their ability both to promote plant growth in other ways and to control phytopathogens that are present in the soil. Biological control agents with resources to make nutrients available to plants are increasingly being targeted by studies^66,68^. In this context, the genus *Trichoderma* comprises fungi of great importance in agriculture; these fungi are known as disease control agents for various pathogens and act as growth promoters of various crop species^28,42,71,72^.

## Conclusions

In this study, *Trichoderma* isolated from soils of the Amazon rainforest demonstrated the potential for phosphate solubilization and increased soybean plant growth, highlighting the importance of the Amazon biome as a source of novel microbial stains with biotechnological importance. The fungus, applied in conjunction with a phosphorus source, promoted soybean plant growth. The two phosphorus sources evaluated in this study showed higher positive effects when combined with the *Trichoderma* isolates than when applied alone. These effects were also P level dependent. Treatments involving different *Trichoderma* strains with beneficial attributes, including the promotion of plant growth and the biocontrol of phytopathogens, should be considered in the development of formulations.

## Materials and Methods

### Collection sites and isolation of *Trichoderma*

**Figure 7:**
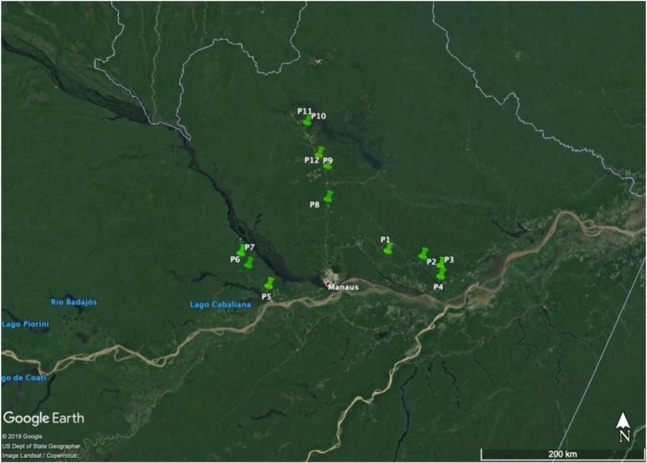
Places for collecting soil samples from the 12 points in the State of Amazonas. From: Phosphorus-solubilizing *Trichoderma* spp. from Amazon soils improve soybean plant growth.

The soil collections were carried out in the State of Amazonas, Brazil, from the city of Manaus, extending to the cities of Itacoatiara, Novo Airão, and Presidente Figueiredo. In total, there were twelve collection points, with a distance between the points from 50 to 60 kilometers, containing three sub-samples per point, collected from 0–15 cm depth. The data of the characteristics of each point are shown in Fig. 7 and Table 1.

For the isolation of *Trichoderma* spp., the selective medium TSM for *Trichoderma*^73^ was used. The soil suspension (1 gr. of soil in 9 mL of sterile saline) was serially diluted and appropriated dilutions were spread plated on TSM medium. The cultures were incubated at 28 °C ± 2 °C for seven days, and after this period, typical *Trichoderma* colonies were purified and selected for further studies. The identification of isolates was performed according to morphological characteristics of the genus *Trichoderma*, by means of colony coloration, characteristics of spores and hyphae.

### Screening of efficient phosphate-solubilizing *Trichoderma* spp

We evaluated the potential of 251 *Trichoderma* isolates capable of solubilize and mineralize P *in vitro*. The isolates were initially grown in potato dextrose agar (PDA) and, later, in solid NBRIP medium (National Botanical Research Institute’s Phosphate) containing 10 g of glucose; 5 g Ca_5_ (OH) (PO_4_)^3^; 5 g MgCl_2_ 6H_2_O; 0.25 g MgSO_4_7H_2_O; 0.2 g KCl; 0.1 g (NH_4_)^2^ SO_4_; 15 g agar and pH 7.0 in 1000 mL distilled water^74^. The plates were incubated at 28 °C ± 2 °C until the presence of a clear hydrolysis halo around the colonies, confirming the ability of the fungus to solubilize P^75^. *Trichoderma* spp. isolates with the highest solubilization halos were evaluated for the quantification of solubilized P in liquid NBRIP medium. The isolates were grown in PDA medium at 28 °C ± 2 °C for seven days. After this period, three 8.0 mm diameter discs were removed and transferred to a 50 mL Erlenmeyer, containing NBRIP medium (glucose, 10 grams (g); MgCl_2_. 6H_2_O, 5 g; MgSO_4_.7H_2_O, 0.25 g; KCl, 0.2 g; (NH_4_)2SO_4_, 0.1 g). In the media, 50 mL of K_2_HPO_4_ (10%) and 100 mL of CaCl_2_ (10%) were added to form an insoluble calcium phosphate precipitate (CaHPO_4_), incubated at 27 ± 2 °C in an orbital shaker at 150 rpm for ten days. The amount of phosphate in the medium before inoculation of the *Trichoderma* strains was approximately 2 µg. mL^−1^ calcium phosphate. Readings were taken at 0, 2, 4, 6, 8 and 10 days. Aliquots of 1 mL were removed and centrifuged at 10,000 g for 5 min to determine the concentration of soluble phosphorus according to the colorimetric method of Murphy & Riley^76^. To evaluate the mineralization potential of selected isolates for liquid NBRIP it was applied a Phytate Specific Media (PSM), containing 15 g C_6_H_12_O_6_; 5 g (NH_4_)_2_SO_4_, 0.1 g NaCl; 0.5 g KCl; 0.01 g FeSO_4_.7H_2_O; 0.01 g de MgSO_4_7H_2_O; 0.01 g MnSO_4_; 5 g calcium phytate and 15 g agar^77^. The plates were incubated at 28 °C ± 2 °C until the presence of a clear hydrolysis halo around the colonies, confirming the ability of the fungus to mineralise P.

The potential for organic acid production was evaluated in high-performance liquid chromatography (HPLC). Aliquots of the samples with 10 days of incubation were collected and centrifuged at 10,000 g for 5 min and filtered in Millipore® 0.2 µm membrane. The extract was applied to a Bio-Rad aminex HPX-87H column, with 10.8% acetonitrile mobile phase at 0.0035 M H_2_SO_4_, and a constant outflow of 0.5 ml min^−1^, 35 °C, UV (210 nm) for 35 minutes.

### Experimental design and bioassay

Soybean plants, variety NA 5909 RG, Brazil, were grown in three-liter pots using soil from the EMBRAPA Environment experimental area, Jaguariúna, São Paulo, Brazil. In the bioassay, a factorial model was applied, including the following factors: two sources of P (Bayóvar rock phosphate and Triple superphosphate), four levels of phosphates (0, 50, 70 and 90 kg ha^−1^), and the application of two *Trichoderma* sp. (AMS 34.39 and AMS 31.15). The control treatment consisted of all levels of phosphates in the two sources without the presence of *Trichoderma*. Twenty-four treatments were applied to the bioassay with five repetitions, counting 120 pots. The two *Trichoderma* isolates used in the bioassay were selected in the *in vitro* test in liquid medium and by a soybean germination bioassay, in order to evaluate if the isolates did not inhibit the germination of the culture used. The soil used in the experiment is characterized by being deficient in P and acid pH, as shown in Table 4.

**Table 4:** Soil chemical analysis.

pH (CaCl_2_)	M.O (g.dm^−3^)	P_resina_ (mg.dm^−3^)	K ----------	Ca ----------	Mg mmolc.dm^−3^	H + Al ----------	SB ----------	CTC ----------	V %
6.0	32	6	<0.9	44	12	<2	56.5	71.5	79

From: Phosphorus-solubilizing *Trichoderma* spp. from Amazon soils improve soybean plant growth.

Phosphorus levels were corrected according to Boletim 100 of the Agronomic Institute of Campinas, São Paulo, Brazil, by means of chemical analysis of the soil according to the crop evaluated. Four levels were assigned to the experiment: R1 or S1, only the phosphorus present in the soil, R2 or S2, R3 or S3 and R4 or S4 being 50, 70 and 90 kg ha^−1^, corresponding to low, medium and high productivity of soybean cultivation, respectively. The proportion of P_2_O_5_ from each of the two sources used was considered, Triple superphosphate (46% of P_2_O_5_) and Bayóvar Rock phosphate (31% of P_2_O_5_). The experiment was conducted for seven weeks until the R1 stage of the culture, under controlled conditions in the wandering house, temperature (25–35 °C), humidity (75–80%) and photoperiod of 10 h/14 h (light/dark). Soil moisture was determined once or two times a day. Bases saturation and pH were corrected with soil liming; and nitrogen and potassium were supplied by irrigating a solution containing 420 mg of urea and 300 mg of potassium chloride in each pot after planting.

### Plant analysis

Twenty-one days after planting, the chlorophyll was measured with a portable SPAD-502Plus meter. At harvest, the height of the soy plants was analyzed. The roots were removed from the soil and washed. The roots were dried (60 °C) until they reached a constant weight for evaluation of the dry matter mass, as well as the leaf area of the plants. The leaves were collected and crushed for subsequent analysis of the P concentration, carried out at the Plant Tissue Laboratory of College of Agriculture “Luiz de Queiroz”, University of São Paulo, Piracicaba, São Paulo- Brazil. The rhizospheric soil was collected for enzymatic analysis of acid and alkaline phosphatases^78^ and phytase^79^.

### Data analysis and statistics

All tests and treatments were performed with repetitions and the values were expressed as the mean between them. For the *in vitro* tests, a parametric variance test (ANOVA) was used to evaluate whether there was a significant difference in the solubilization of P, after considering the assumptions of normality tested by the Shapiro-Wilk and equality of variance by bartlett test. The significant data were compared using the Tukey and Scott Knott test (p < 0.05).

In the greenhouse experiment, a two-way ANOVA was applied to test the significance of each factor (levels of phosphorus and *Trichoderma* ssp.) and its interaction. As the interactions were always significant, Scott Knott mean comparation test was applied for the treatments considering the P levels, the *Trichoderma* isolates, and the control treatment.

To measure the effectiveness of the addition of *Trichoderma* in each level of P and the two sources of P, absolute values of dry weight (DW) (g/plant-1) were converted in the improvement of the biomass of the plants (in %) for each *Trichoderma* sp. calculated in relation to the control without Trichoderma. For that, we applied the following Eq. 1:

1 $$Improvement\,( \% )=\frac{(Trichoderma\,x;Level\,y\,\ast \,100)}{Control;Level\,y}$$

where each of the isolates of *Trichoderma* (x) - AMS 34.39 and AMS 31.15 - at each level of phosphorus (y) - 0, 50, 70 and 90 kg ha^−1^ - is compared with the control conditions at the same levels of P (y). The value different from 0% indicates that treatment with *Trichoderma* resulted in an increase or decrease in plant biomass (using the same source of P and the level applied). The amount of phosphorus in the aerial part of the soybean plants was evaluated between the *Trichoderma* and control isolates, in relation to the source of phosphorus and level of this applied. This value was obtained by multiplying the phosphorus content of the aerial part of the plant by its dry matter. In addition, the efficiency of P absorption (in %) between the phosphorus sources and the applied level was calculated by the Eq. 2:

2 $$Efficiency\,( \% )=\frac{(Trichoderma\,x;Level\,y-Control;Level1)}{(Control;Level\,y-Control;Level1)}$$

where each of the isolates of *Trichoderma* (x) – AMS 34.39 and AMS 31.15 – at each level of phosphorus (y) - 0, 50, 70 and 90 kg ha-1 – and the control conditions at the same levels of P (y) were compared with the control without the addition of phosphorus- control Level 1.

## Data Availability

The datasets generated during and/or analysed during the current study are available from the corresponding author on reasonable request.
